# Epidemiological study of prevalent pathogens in the Northwest region of Argentina (NWA)

**DOI:** 10.1371/journal.pone.0240404

**Published:** 2020-10-13

**Authors:** Mónica Florencia Torrez Lamberti, Fabián Enrique López, Patricia Valdez, Ana Bianchi, Evangelina Barrionuevo Medina, María de las Mercedes Pescaretti, Mónica Alejandra Delgado

**Affiliations:** 1 Instituto Superior de Investigaciones Biológicas (INSIBIO), CONICET-UNT, and Instituto de Química Biológica “Dr. Bernabé Bloj”, Facultad de Bioquímica, Química y Farmacia, UNT, San Miguel de Tucumán, Argentina; 2 Hospital Interzonal de Niños Eva Perón (HINEP), San Fernando del Valle de Catamarca, Catamarca, Argentina; 3 Centro Provincial de Salud Infantil “Eva Perón” (CePSI), Santiago del Estero, Argentina; 4 Hospital del Niño Jesús (HNJ), San Miguel de Tucumán, Tucumán, Argentina; Nitte University, INDIA

## Abstract

Northwest Argentina (NWA) is a poor economic-geographical region, with the highest rate of diarrhea diseases. At the moment, there are no reports showing the epidemiological status of this region that would allow to establish methods for prevention and control of these infections and to indicate of the prevalent pathogen that produces them. Therefore we carried out an epidemiological study of the gastroenteritis etiological agents and their incidence in the pediatric population. A total of 17 823 fecal samples were collected, 14 242 from HNJ-Tuc, 2,257 from CePSI-Stgo and 1,324 from HINEP-Cat. In 2,595 samples a bacterial agent was identified, the 93.64% corresponded to *Shigella*/*Salmonella* clinical isolates. *Shigella* genus was the prevalent pathogen, being *Shigella flexneri* 2 the most frequent serotype. Most of the *Shigella* clinical isolates presented themselves as multidrug-resistant (MDR), harboring 2 to 3 genetic resistance determinants. 50% of the affected patients were children under 4 years old. Here, we demonstrate that bacterial gastrointestinal diseases strongly affect the health of NWA population. The appearance of epidemic outbreaks, as happened during 2014, suggest that they may be related to the socio-economic poverty of NWA. Recently, *Shigella flexneri* 2 has become the highest NWA´s incidence infectious agent. The acquisition of new antibiotic resistance determinants may play an important role in their adaptation and persistence.

## Introduction

Infectious diseases causing diarrhea are among the major cause of morbidity and mortality in children under 5 years old [1–3]. Foodborne Diseases (FBD) are transmitted by consumption of contaminated food. *Escherichia coli* (*E*. *coli*), *Salmonella*, and *Shigella* are the principal cause of most of the worldwide foodborne outbreaks [4–7].

According to the World Health Organization in America around 77 000 000 people are affected by FBD, causing about 2,000 deaths of children under 5 years old per year [8, 9]. In Argentina shigellosis is one of the main causes of children’s morbidity and mortality, where around 1 200 000 cases of acute diarrhea in public hospitals are being reported per year [10]. NWA is an economic-geographical region that involves 6 of the 23 provinces that establish the Argentine territory [11]. The acute diarrhea cases of NWA represent the 30% of the total Argentina’s cases [10].

In Argentina, *Shigella* is the major causative agent of diarrhea, followed by *Salmonella* and *E*. *coli* strains [12–14]. The aim of this work was to conduct the first epidemiologic study of bacterial pathogens causing gastrointestinal disease, determining the incidence of *Salmonella* and *Shigella* in the pediatric population of three provinces of NWA.

## Materials and methods

### Study area and period

In this study we collaborated with the hospital from Catamarca (Hospital Interzonal del Niño "Eva Perón", HINEP), Santiago del Estero (Centro Provincial de Salud Infantil "Eva Perón", CePSI) and Tucumán (Hospital del Niño Jesus, HNJ). These hospitals are the central emergency department responsible for receiving and processing most fecal samples (Fs) from patients suffering gastroenteric diseases. This study included the systematic collection of Fs during the 2013–2017 period, from patients up to 14 years old. This manuscript has been approved by the appropriate ethical committees of the Ministerio de Salud Pública de la Provincia de Tucumán (Sección de Investigación en Salud-SIPROSA, Expediente N° 1134-410D-2019); Administración Nacional de Medicamentos, Alimentos y Tecnología Médica-Argentina (ANMAT) and the Registro Nacional de Investigaciones Sanitarias (ReNIS), which are related to the institutions (Public Hospitals) where it was performed. All participating members gave their informed consent as confidential data of each hospital.

### Sample collection and pathogen identification methods

The stool samples were placed into sterile plastic containers, were maintained in Cary Blair medium and and transported to the laboratory. They were processed immediately after arrival, using the stool test. The samples were directly seeded in enrichment and selective/differential culture media plates as described in S1 Fig [15–18]. The different clinical isolates (CI) were identified using the Hospitals’ standard biochemical tests as was described [15–18].

### Clinical isolates classification

All CIs obtained were classified by species and serotype levels with an antigenic analysis. The slide agglutination technique was carried out using specific sera obtained from ANLIS‐Malbrán.

The identification of *S*. *flexneri* and *S*. *sonnei* was prevalent. This was confirmed by multiplex PCR (mPCR) as was described [19]. The PCR products obtained from 10 CIs of each *Shigella* serotype were detected by electrophoresis in 1.2% agarose gels stained with ethidium bromide.

### Antimicrobial susceptibility and plasmid analysis

Antimicrobial susceptibility tests were performed on Oxoid Müller–Hinton agar by the disk diffusion method. The antibiotics used were: ampicillin (Amp, 10 μg), chloramphenicol (Cm, 30 μg), kanamycin (Km, 30 μg), fosfomycin (200 μg), furazolidone (300 μg) and trimethoprim sulfamethoxazole (TMS, 1.25/23.75 μg). The antimicrobial susceptibility testing standards were performed following the CLSI recommendations. Resistance and sensitivity were interpreted according to the CLSI criteria [20].

The plasmid extraction was performed by the alkaline lysis method as was described [21]. The plasmids were analyzed by electrophoresis on agarose gels, stained with ethidium bromide and UV light exposition. Plasmid molecular size estimation was conducted by calibration curves using the Supercoiled Plasmidic DNA molecular marker (Promega).

## Results

### Gastrointestinal diseases incidence in the pediatric population of three NWA provinces

**Fig 1:**
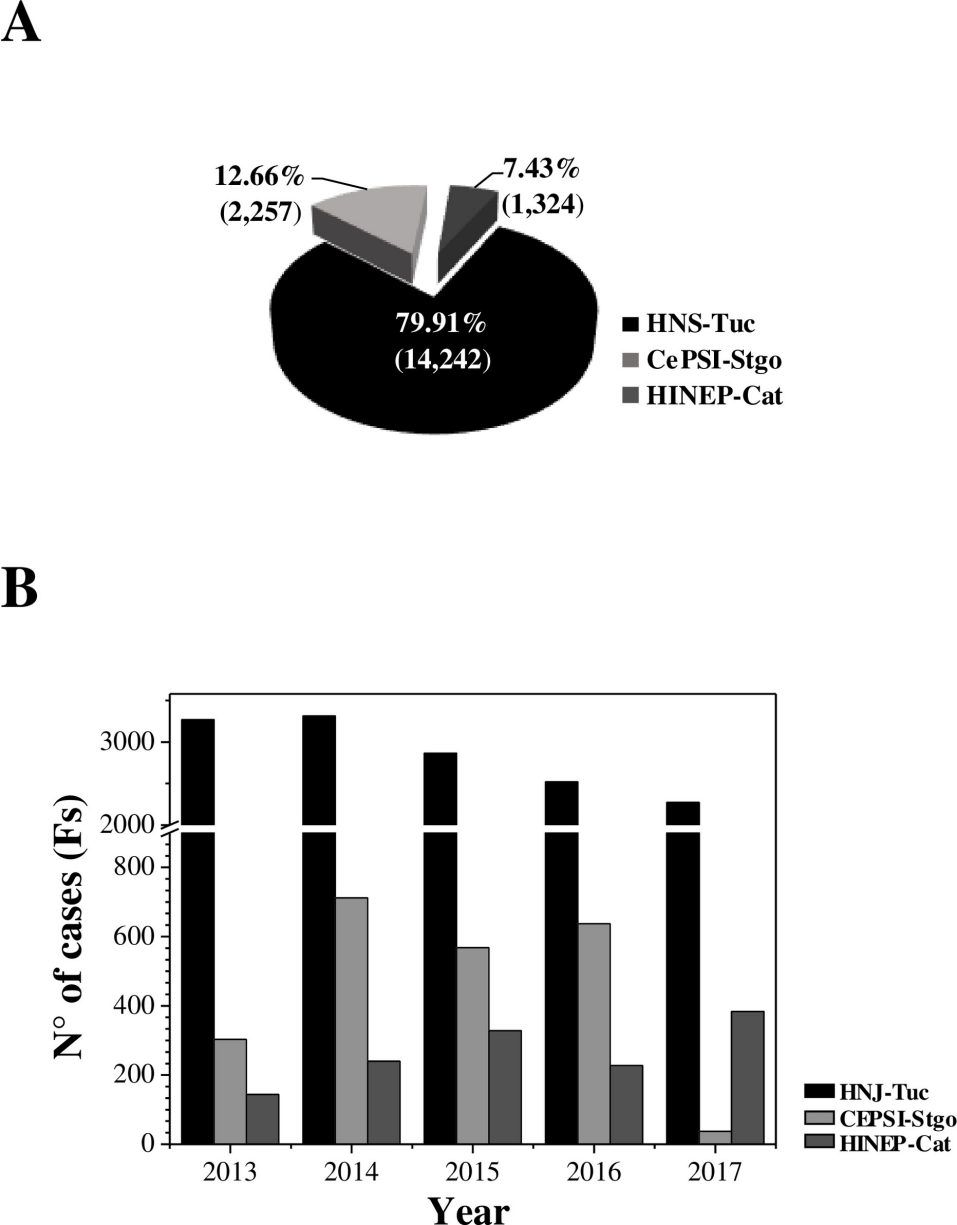
Isolation rates and incidence of infection disease agents in the NWA region´s Hospitals. (A) Percentage of fecal samples (Fs) by Hospital, numbers of isolates are in parenthesis. (B) Cases numbers obtained per year in each Hospital.

A total of 17 823 Fs from patients under 14 years old suffering acute diarrhea from three NWA Hospitals were collected and analyzed: 2,257 Fs from CePSI-Stgo; 1,324 from HINEP-Cat and 14 242 from HNJ-Tuc. The highest number of isolates (79.91% of the total) was registered in HNJ-Tuc, indicating that this population was the most affected (Fig 1A). Meanwhile the 12.66% and 7.43% were from CePSI-Stgo and HINEP-Cat, respectively (Fig 1A). In concordance with the size of the pediatric population estimated by Argentina’s INDEC [22, 23], we observed that the incidence of diarrheal diseases in Tucumán´s population was 33.01 of the total average. These results suggest that 33.01 children from 1,000 suffered at least one diarrheal disease episode during the analyzed period (Table 1). Conversely in Santiago del Estero and Catamarca the incidence was 8.5 and 12.6, respectively (Table 1). The pediatric population of the three provinces did not have major changes throughout the studied years (Table 1) [22, 23]. These data indicate that Tucumán displays 2 to 4-fold increased pediatric population than Santiago del Estero and Catamarca, respectively. Interestingly, when the incidence of diarrheal disease per year was analyzed in each province, a sharp decrease for 2015 was observed in Tucumán, while in the other provinces it remained relatively constant or weakly increased (Table 1).

**Table 1:** Incidence of gastroenteric disease in three provinces of NWA.

Area	Year	Population under 14 years old*	Cases	Incidence**
**HNJ-Tuc**	2013	428 936	3,269	7.62
	2014	429 937	3,314	7.70
	2015	431 166	2,866	6.64
	2016	432 657	2,519	5.82
	2017	434 416	2,274	5.23
**Total/Average**		**431 422.4**	**14 242**	**33.011**
**CePSI-Stgo**	2013	270 090	303	1.12
	2014	267 789	712	2.65
	2015	265 841	568	2.13
	2016	264 329	637	2.40
	2017	263 288	37	0.14
**Total/Average**		**266 267.4**	**2,257**	**8.47**
**HINEP-Cat**	2013	107 230	144	1.34
	2014	106 095	240	2.26
	2015	105 043	328	3.12
	2016	104 110	228	2.18
	2017	103 317	384	3.71
**Total/Average**		**105 159**	**1,324**	**12.59**

*The estimated values were taken from Rubinstein, 2018 [10]

**The incidence rate was calculated as number of events divided by the pediatric population (per 1,000), during the observation period [23].

The large number of cases recorded in HNJ-Tuc can be attributed to the high population density. Tucumán is the smallest surface province (22 524 km^2^) harboring 19 children/Km^2^, while Catamarca (102 602 km^2^) and Santiago del Estero (136 351 km^2^) have 1 and 2 children/Km^2^, respectively [10, 24]. Here, we observed that Tucumán has a 9.8- and 18-folds’ greater pediatric density compared to Santiago del Estero and Catamarca. The density levels were not correlated with the number of samples processed per year. The total number of Fs in HNJ-Tuc was, while in CePSI-Stgo remained high during 2014–2016; and in HINEP-Cat a marked increase was observed through the whole period (Fig 1B).

### Enteric pathogens prevalence in the NWA region

Standard biochemical and microbiological diagnostics were performed to partially identify the bacterial agent present in the 17 823 Fs. Meanwhile, serological classification for species level was conducted by sera-specific tests. The results showed that in 14.55% (2,595 Fs) of the total studied cases at least one bacterial genus was isolated. The 93.64% of this 2,595 CI corresponded to the genus *Shigella* and *Salmonella*, placing them as the most common pathogens isolated (Table 2). Whereas in the remaining 6.36% (165 CI) others bacterial species such as *Vibrio* spp, *Aeromona* spp, *E*. *coli* O157:H7 and *Campylobacter* were identified (Table 2). We also observed that *Salmonella* had a lower occurrence than *Shigella* (6.96% vs 93.04%, Table 2) in the three hospitals. Interestingly, when the number of pathogens isolated was compared among the geographical areas, we found a higher percentage of *Salmonella* sp. (12.40%) in HINEP-Cat than in CEPSI-Stgo (2.5%) or HNJ-Tuc (6.92%) (Table 2).

**Table 2:** Identification of bacterial agents causing gastroenteric disease in three provinces of NWA.

Hospital/Year	Total N° of cases	N° of Clinical Isolates	Identified bacterial genus		
			*Shigella*	*Salmonella*	Other
**HNJ-Tuc**					
2013	3269	312	288	13	11
2014	3314	439	367	34	38
2015	2866	397	353	21	23
2016	2519	443	388	22	33
2017	2274	361	278	26	57
**Total**	**14 242**	**1,952**	**1,674**	**116**	**162**
**CePSI-Stgo**					
2013	303	40	38	2	0
2014	712	78	78	0	0
2015	568	33	33	0	0
2016	637	47	44	3	0
2017	37	7	7	0	0
**Total**	**2,257**	**205**	**200**	**5**	**0**
**HINEP-Cat**					
2013	144	40	37	3	0
2014	240	139	132	7	0
2015	328	108	88	19	1
2016	228	72	59	12	1
2017	384	79	71	7	1
**Total**	1,324	438	387	48	3
**TOTAL**	**17 823**	**2,595**	**2,261**	**169**	**165**

**Fig 2:**
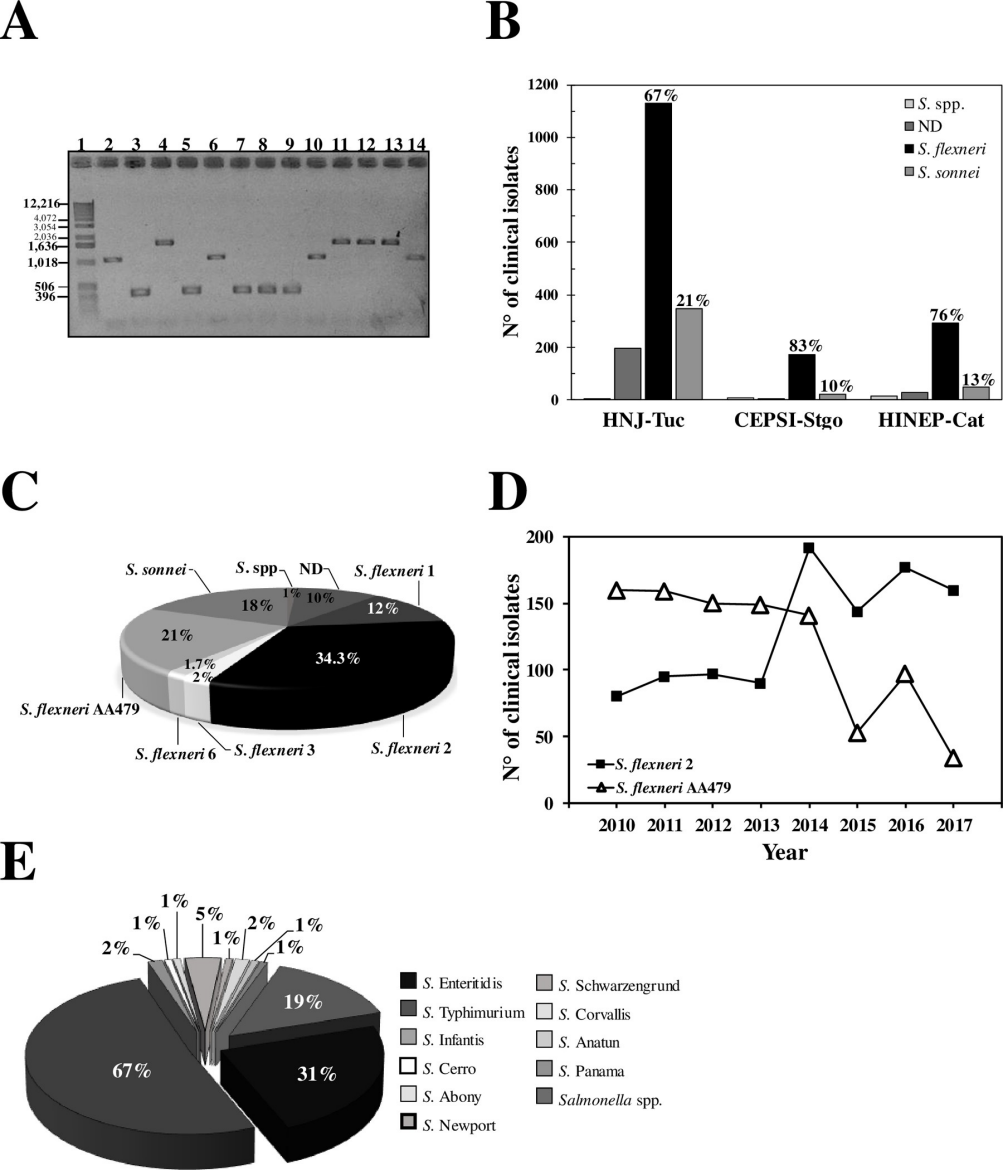
Enteric bacteria incidence in the NWA´s province during the 2013–2017 period. (A) Identification of *S*. *flexneri* and *S*. *sonnei* by molecular tools (mPCR). PCR products were analyzed by electrophoresis in 1.2% agarose, representative gel: 1- 1Kb Molecular marker: 2- *S*. *sonnei* (control); 3- *S*. *flexneri* non 2a (control); 4- *S*. *flexneri 2a* (control); 5- AC147;6- AC148; 7- AC152; 8- AC156; 9- AC159; 10- AC160; 11- AC161; 12- AC162; 13- AC163 and 14- AC168. (B) Number of *Shigella* clinical isolates identified per year in each Hospitals of the NWA region. (C) Incidence of the different *S*. *flexneri*´s serotypes isolated in the region. (D) Changes in the prevalence of *Shigella* serotypes in the NWA region, in recent years. (E) Incidence of the different *Salmonella enteritidis* serotypes identified during the study period.

We selected 10 CIs of each *Shigella* serotype from each hospital per year to confirm the serological classification using mPCR. In concordance with Farfán *et al*. (2010), the serum specific test results matched with the mPCR profiles (Fig 2A). These results demonstrated that *Shigella* genus is the prevalent pathogen in the study area, where *S*. *flexneri* was the most abundant specie (67 to 83% of CI) followed by *S*. *sonnei* (10 to 21%) (Fig 2B). In the three hospitals only 5 of the 14 *S*. *flexneri* serotypes were identified, being *S*. *flexneri* 2 (34.3%) the most frequent followed by *S*. *flexneri* AA479 (21%) and *S*. *flexneri* 1 (12%) (Fig 2C).

Using the database records from previous years (since 2010) in each hospital, we analyzed the *S*. *flexneri* AA479 and *S*. *flexneri* 2´s incidence. We found that *S*. *flexneri* AA479 was the prevalent pathogen in preceding years, but its prevalence drastically decreased over the years while the number of *S*. *flexneri* 2 isolations were increasing (Fig 2D).

As mentioned, *Salmonella* spp. was mainly isolated in Catamarca and Tucumán (Table 2). The serological analysis showed that only 10 different serovars of *Salmonella enterica* (*S*. *enterica*) were present in this region, being Typhimurium (*S*. Typhimurium, 67%) and Enteritidis (*S*. Enteritidis, 31%) serovars the prevalent ones (Fig 2E).

### Geographical distribution of the studied pathogens

**Fig 3:**
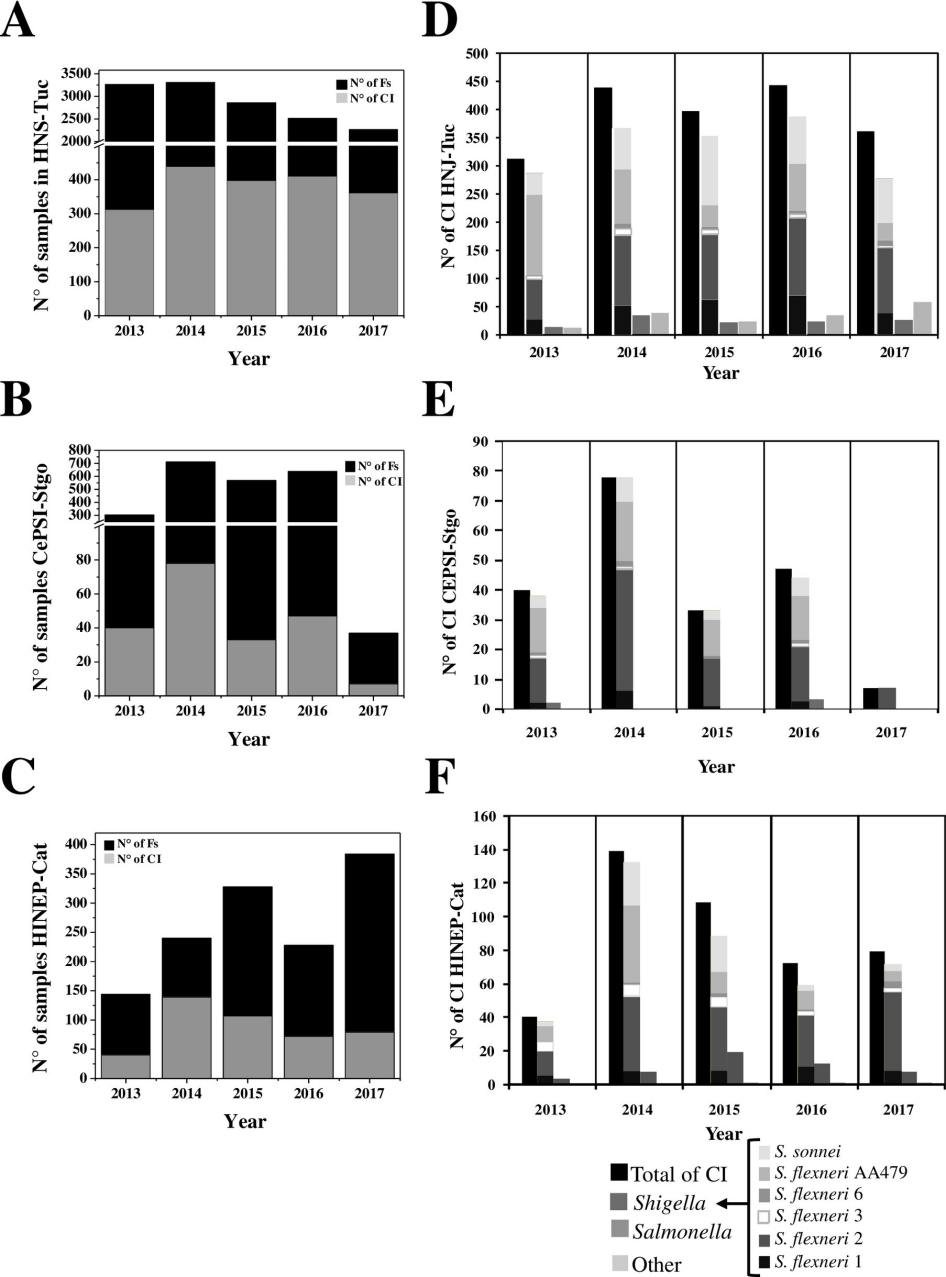
Ration of the bacterial CI´s number per year. Ration of the bacterial CI´s number per year was related to the number of fecal samples obtained (A, B, C) and distribution of the pathogen genus isolated in each Fs (D, E, F) of the NWA´s Hospitals: HNS of Tucumán (A, D), CePSI of Santiago del Estero (B, E) and HINEP of Catamarca (C, F).

In Argentina the 30% of the bacterial infections reported affect the NWA [10]. We observed that 14.5% of the total Fs studied were caused by bacteria (Table 2). The incidence of the bacterial pathogen was analyzed considering its origin and the year of study. We observed that the number of CI in HNJ-Tuc remained relatively constant at high levels, while in the rest of the provinces these levels tended to decrease from 2015 (Fig 3A–3C). Moreover, we found that even when in HINEP-Cat a smaller number of Fs were processed, the number of CI was 2 folds higher than in CePSI-Stgo. In addition, during 2014 there was an increase in the number of CI in the three-sample collection areas, suggesting a possible epidemiological outbreak. We observed that the incidence of *Shigella* infections was the highest during 2014 (84–98%), since *Shigella* isolates outweighed *Salmonella* or other bacterial species isolations in all the analyzed places (Fig 3D–3F). Although only 2 of the 4 species of *Shigella* (*S*. *flexneri* and *S*. *sonnei*) were detected, the diversity of *Shigella* serovars was not homogeneous over the studied hospitals, and only 5 of 14 *S*. *flexneri* serovars were identified. Moreover, throughout the period analyzed in these places the increased persistence of *S*. *flexneri* 2 and the decrease of *S*. *flexneri* AA479 isolations remained constant. Remarkably, in HNJ-Tuc a high percentage *S*. *sonnei* (20–35%) was identified (Fig 3D).

### Age range affected by the studied pathogens

**Fig 4:**
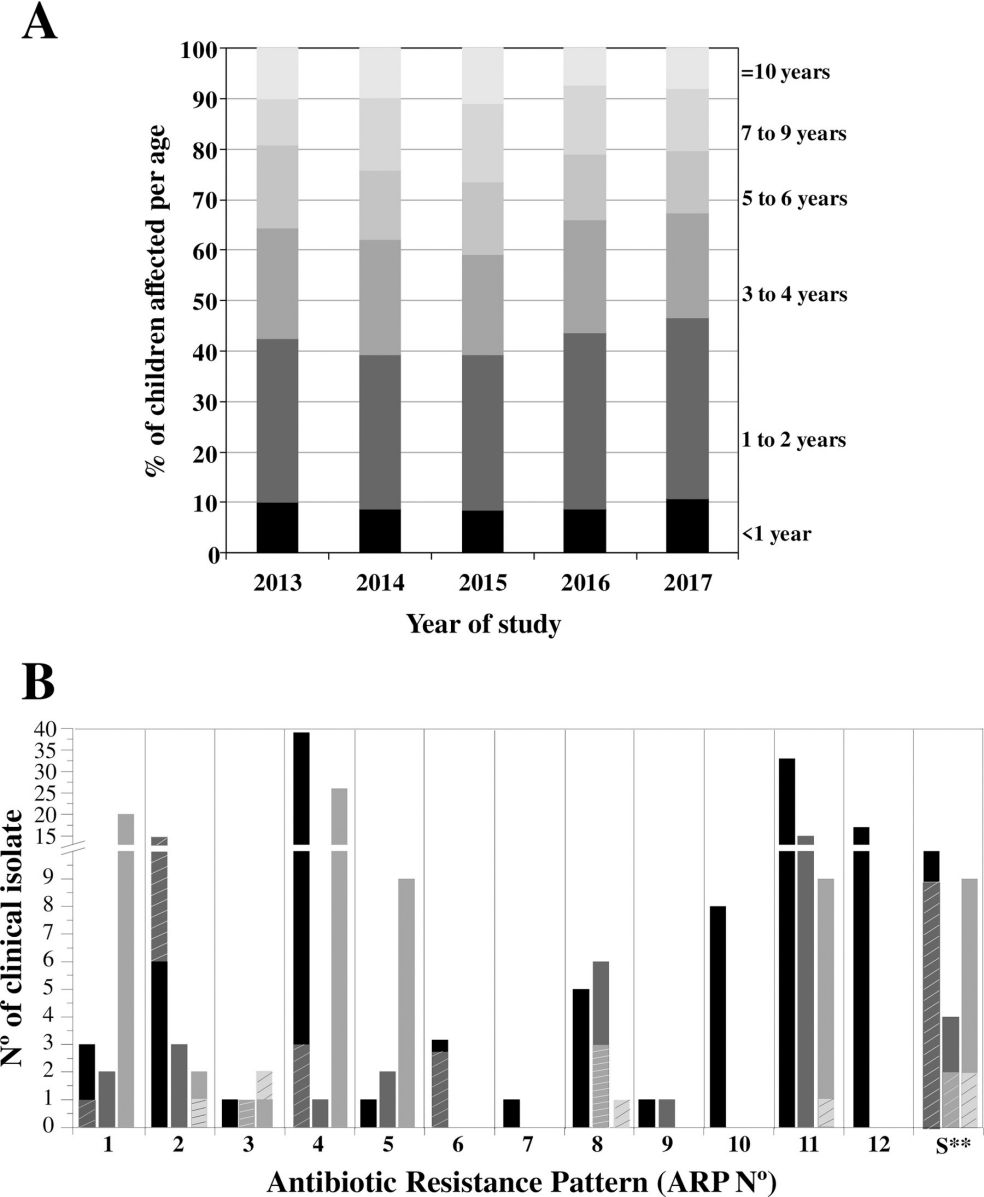
Distribution of the CIs according to the age of the patients and to the antibiotic resistance. (A) Age distribution of the patients affected by gastrointestinal diseases during the 2013–2017 period, analyzing and taking into account the total number of patients from each NWA´s Hospitals. (B) Antibiotic Resistance Pattern (ARP). The antibiotic resistance of *S*. *flexneri* (full bar) and *S*. *sonnei* (striped bar) CIs obtained from HNJ-Tuc (black bar), CePSI-Stgo (dark gray bar) and HINEP-Cat (gray bar) was determined as described [16]. Twelve different patterns were obtained: ARP1 (Cm), ARP2 (Amp), ARP3 (TMS), ARP4 (Amp Cm), ARP5 (TMS Cm), ARP6 (Amp Km), ARP7 (Cm Km), ARP8 (TMS Amp), ARP9 (TMS Amp Km), ARP10 (Amp Cm Km), ARP11 (TMS Amp Cm) and ARP12 (TMS Amp Cm Km). S**: N° of CI sensitive to all antibiotics tested.

In this study a range of age from newborns up to 14 years old was considered. We determined that the highest percentage of patients suffering such infection (around 50%) belonged to the age range between 1 to 4 years old (Fig 4A). However, it was also observed that 30% of the cases corresponds to a range of age of 5 to 9 years old, while children above 9 or younger than 1 were less susceptible to acquire the disease (10% each group) (Fig 4A).

### Antimicrobial susceptibility pattern of the isolated pathogens

For a deeper analysis, we selected and stored 320 CIs, maintaining the ratio of *Shigella* and *Salmonella’s* serovars obtained per year and hospital (283 and 37 CIs, respectively). To characterize these CIs, antibiotic standard susceptibility tests were performed following the CLSI recommendations [20]. The results showed that 100% of *Salmonella* CI were sensitive to all the antibiotics analyzed, in contrast to 13% of 283 *Shigella* CIs were sensitive (Fig 4B). The remaining 87% of *Shigella* CIs were clustered into 12 different antibiotic resistance patterns (ARP) of 36 putative combinations (Fig 4B).

In HNJ-Tuc 12 different ARPs were presented, containing at least one *S*. *flexneri* CI (Fig 4B). The MDR-ARP-4 was the most abundant group followed by ARP-11 and ARP-12. In CePSI-Stgo the ARP-11 included the largest number of CI, while in HINEP-Cat the ARP-4 and ARP-1 patterns were prevalent. Almost all the *S*. *sonnei* CIs displayed non antimicrobial resistance, except in HNJ-Tuc where the highest number of *S*. *sonnei* was Amp resistant. These results showed that the prevalent pathogenic strains presented themselves as MDR bacteria, containing between 2 and 3 genetic determinants of resistance. Interestingly, the Amp resistance was the most widely distributed among these pathogens regardless of place and year of isolation.

We also analyzed the plasmid content of the CIs belonging to the most persistent ARP groups. Using a molecular marker, we were able to infer the number and size of the putative plasmids showing a great variety of them (Table 3). We observed that the great diversity in plasmid content is present not only inter *S*. *flexneri* and *S*. *sonnei* but also intra species (Table 3). As shown in Table 3, the wide diversity of plasmids was fully distributed over each ARP groups. Two to three plasmid profiles were found among the multidrug-resistant strains of *S*. *flexneri*, which were independent of the place and the year of isolation. Among the *S*. *sonnei* isolates selected according to their antimicrobial susceptibility group, two plasmid profiles sharing a plasmid of approximately 6.0 kb were observed.

**Table 3:** Plasmid content of *Shigella* clinical isolates belonging to the most frequent antimicrobial resistance profiles.

Specie	ARP N°	Antibiotic resistances	Plasmid profile (approximate MW in Kb)		
			I^a^	II^a^	III^a^
***S*. *flexneri***	**1**	Cm	8; 5; 3,2	5; 2	5
	**2**	Amp	7,5; 5,5; 4,8; 4,2; 2,8; 1,5	5,5; 2,8	2,8
	**4**	Amp Cm	6; 5; 3; 2; 1,5	6; 2	2; 1,5
	**5**	TMS Cm	6,8; 5; 4,2; 3,4; 3; 2,8	6,8; 5; 4,2; 3,4	5; 3,7
	**8**	TMS Amp	7; 4,8; 3; 2,2	5,5; 4,5; 3,5	5,5
	**11**	TMS Amp Cm	10; 8; 5,8	10; 7; 4	---
	**12**	TMS Amp Cm Km	3,5; 3; 2,5; 1	4	---
***S*. *sonnei***	**2**	Amp	8; 6; 3	8; 6; 2,5	---
	**4**	Amp Cm	8; 6; 5, 1; 2	---	---
	**8**	TMS Amp	6; 4,9; 3,2	8; 6	---

^**a**^The number and size of plasmids for each profile was estimated based on the pattern displayed by each CI in an agarose gel, using a calibration curve detailed in Methods.

## Discussion

Gastroenteric infections are among the main causes of morbidity and mortality of pediatric population [9]. The number of reported cases puts Argentina at the same level as Cuba and 307.5% higher than Chile [10]. We carried out an epidemiology study to determine the gastroenteritis incidence in NWA pediatric population, since there were no reports showing the epidemiological status of this region. When the average of these province’s population size [11, 22] was considered, we observed that 2.2% of children suffered at least one diarrhea episode during this period, where 11.3% of the total were produced by some bacterial agent. In this period there was an increase in the number of patients with gastroenteritis, mainly in HINEP-Cat where this increase was more distinct. Despite the increased number of Fs obtained, the CI number was slightly diminished, except in CePSI-Stgo where a significant reduction was observed. Conversely the pediatric population of Tucumán was the most affected per year, probably due to the high population density concentrated in the urban area of this province. This suggestion was based on the data reported by INDEC 2010, where Tucumán´s population exceeded 1.7 and 4 times the Santiago del Estero and Catamarca populations [11]. In Tucumán the rate of Fs/CI was maintained along the years of study, suggesting that the bacterial infections could not be controlled, at least in this region. We observed that the flow of admitted children in HNJ-Tuc was also higher than in CePSI-Stgo and HINEP-Cat (6.4 and 10-fold respectively), suggesting that this increased flow occurs by person-to-person transmission due to the high population density. Meanwhile, in Santiago del Estero and Catamarca other factors as the economy, hygiene and health conditions may be the major influencers. However, when the pediatric population size and the flow of patients per province were considered it was observed that Catamarca is the province with the highest percentage of positive bacterial cases.

During 2014, the total number of Fs analyzed was greater than the previous year, suggesting that an infectious outbreak could have occurred. These data is correlated with previous reports, in which during 2014 and 2016 the rate of gastrointestinal diseases was around 10 and 40 times higher than the vectorial and Zoonotic Immuno-preventable diseases, respectively [24]. Our results suggest that this outbreak was caused by bacterial agents, since we was also observed a ≅1.4 to 3-fold increase in the number of CI.

In accordance with previous reports we observed that about 10% of the total cases of bacterial infection corresponded to newborns (0 and 1 year old), while the most affected were the children between 1 to 4 years old (~55%) [25–27].

We demonstrated that in the NWA, *Shigella* isolates were more frequent (87.12%) than reported in other Argentina´s regions [28–32]. The presence of *Shigella* is used as an indicator of hygiene level: *S*. *flexneri* is isolated in poorer regions while *S*. *sonnei* is identified in industrialized areas. Our serotyping results indicated that the number of *S*. *flexneri* isolations was higher than *S*. *sonnei*. These results confirm the poverty degree of the NWA region as was reported [33, 34]. The percentage of poor people residing in the NWA is five times bigger than in Buenos Aires city (Argentina`s capital) and almost triple than Southern Patagonia [33, 35]. In addition, we demonstrated that *S*. *flexneri* 2 isolations increased over the number of *S*. *flexneri* AA479, positioning itself as the prevalent pathogen of the NWA region. In concordance, Merino *et al*. reported that *S*. *flexneri* 2 was the prevalent pathogen in the Northeast region of Argentina (NEA) [30].

In this study, *S*. *flexneri* strains isolated showed a multidrug-resistant pattern. However, a high frequency of ampicillin or chloramphenicol resistant isolates was also observed. We can suggest that over time the CIs belonging to ARP-4 (Amp^R^Cm^R^) could sequentially acquire 1 or 2 determinant antibiotic resistance genes (TMS^R^ for ARP-11 and TMS^R^ Km^R^ for ARP-12) to become MDR strains, due to the wide and indiscriminate use of antimicrobial drugs [36, 37]. The identification of *Shigella* MDR isolates was also reported in NEA, but with a different pattern [30]. Despite the fact that we could not find a direct relation between the plasmid patterns and the ARPs, the great diversity observed suggests the horizontal gene transfer as a possible mechanism to spread these resistances among the pathogens affecting the NWA population.

## Supporting information

S1 Fig Steps followed for pathogens isolation from fecal sample. MacConkey: selective/differential medium. XLD agar: selective medium. Salmonella/Shigella agar (SS agar): selective/differential medium. Selenito Broth/Tetrationato: enrichment media. Scheme adapted from the Microbiological Procedure Handbook of Koneman, E.W. and Allen, S. (2008) and Murray, P.R. (2013), routinely used in the Bacteriology´s labs of the Hospitals involved in this study [18].
(PDF)

S1 Raw image (TIF)
