# Supplementary figures and images for: Epidemiological study of prevalent pathogens in the Northwest region of Argentina (NWA)
Source: PLoS One. 2020 Oct 13;15(10):e0240404. doi: 10.1371/journal.pone.0240404 (PMC7553278; doi:10.1371/journal.pone.0240404)

# Supplementary Figure 1

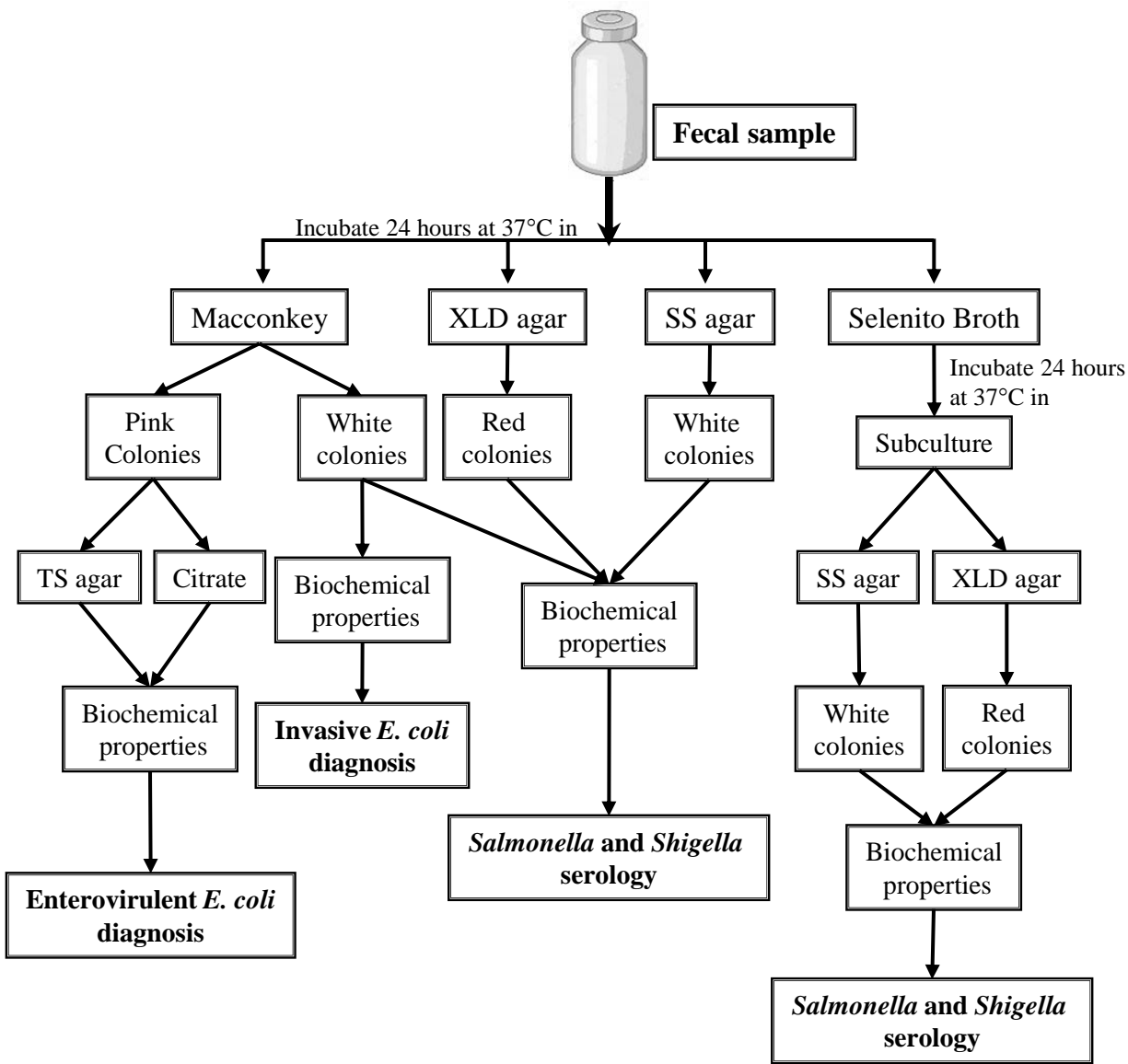

Supplement: S1 Fig — MacConkey: selective/differential medium. XLD agar: selective medium. Salmonella/Shigella agar (SS agar): selective/differential medium. Selenito Broth/Tetrationato: enrichment media. Scheme adapted from the Microbiological Procedure Handbook of Koneman, E.W. and Allen, S. (2008) and Murray, P.R. (2013), routinely used in the Bacteriology´s labs of the Hospitals involved in this study [18]. (PDF) [file pone.0240404.s001.pdf]

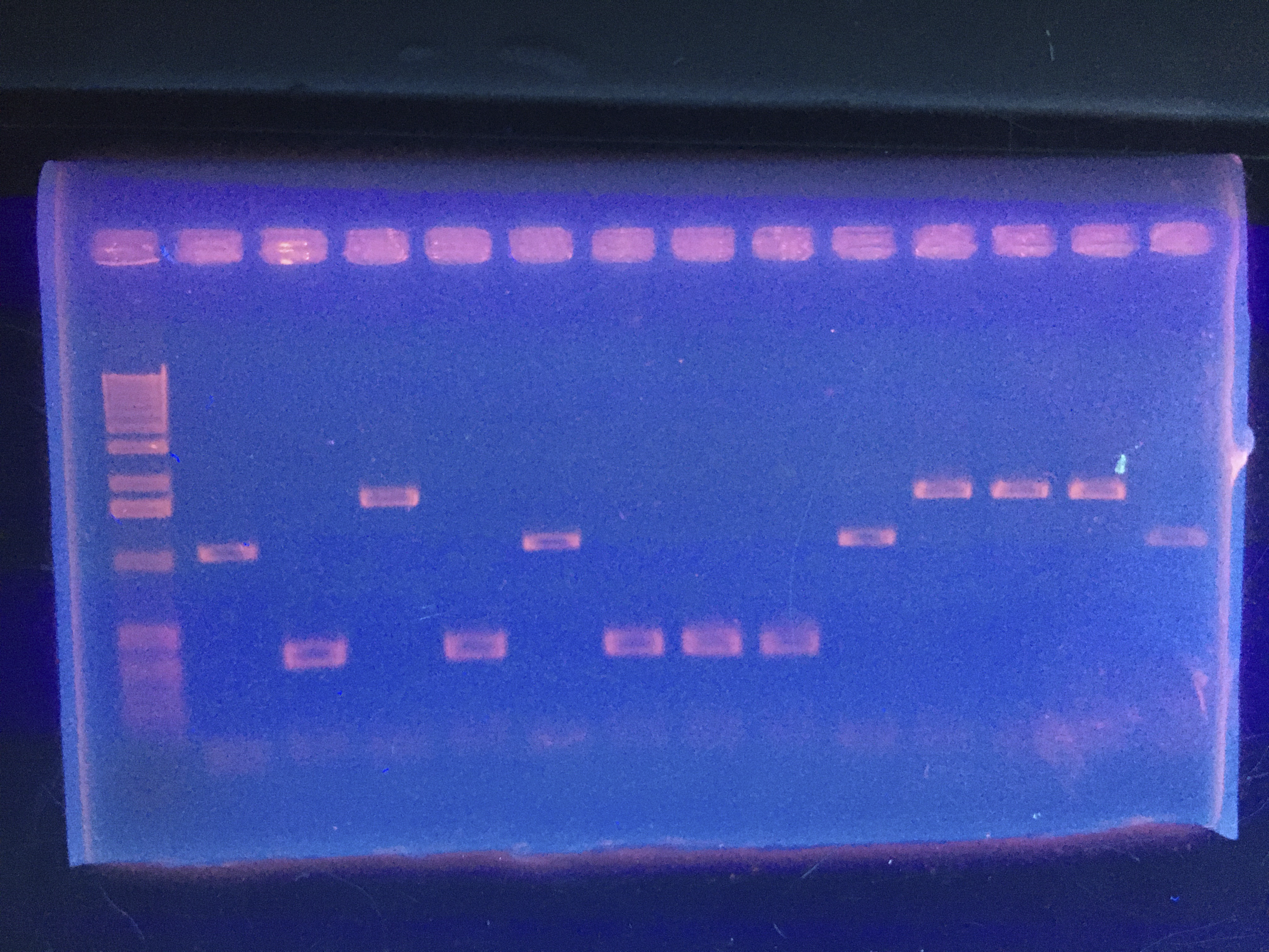

Supplement: S1 Raw image — (TIF) [file pone.0240404.s002.tif]
